# Dystonic Tremor Disappearance after Internal Capsule Stroke

**DOI:** 10.1002/mdc3.13790

**Published:** 2023-05-31

**Authors:** Arthur W.G. Buijink, Anke H. Snijders, Rick C. Helmich

**Affiliations:** ^1^ Department of Neurology Amsterdam University Medical Centers, Amsterdam Neuroscience, University of Amsterdam Amsterdam The Netherlands; ^2^ Department of Neurology, Center of Expertise for Parkinson & Movement Disorders Donders Institute for Brain, Cognition and Behaviour, Radboud University Medical Centre Nijmegen The Netherlands; ^3^ Centre for Cognitive Neuroimaging Donders Institute for Brain, Cognition and Behaviour, Radboud University Nijmegen The Netherlands

**Keywords:** tremor, dystonic tremor, stroke, lesion

Dystonic tremor is defined by the occurrence of tremor and dystonia in the same body part.^1^ Dystonia secondary to stroke is a well‐known phenomenon,^2^ however the disappearance of dystonia or dystonic tremor after stroke has not been reported.^3^ We present a patient with dystonic tremor, which was abolished on the right side after contralateral stroke in the posterior limb of the internal capsule.

## Case Report

**Figure 1:**
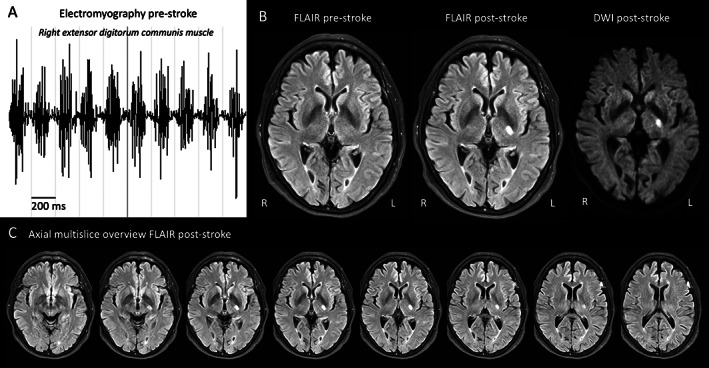
Polymyography and magnetic resonance imaging (MRI) of the brain pre‐ and post‐stroke. (**A**) Surface electromyography shows tremulous with a frequency between 4.3 and 5.6 Hz of right extensor digitorum communis muscle pre‐stroke. (**B**) On the left, a T2‐fluid‐attenuated inversion recovery (FLAIR) sequence pre‐stroke shows no abnormalities. Next a FLAIR sequence post‐stroke showing a hyperintensity in the posterior limb of the internal capsule, in the vascular territory of the anterior choroidal artery, which is confirmed on a diffusion‐weighted image (DWI, b = 1000). (**C**) Axial multislice overview of FLAIR images post‐stroke.

A 64‐year‐old woman was referred to us as a potential candidate for stereotactic surgery for tremor. She experienced shaking of the arms that had progressively worsened over the past six years, without sufficient effect of primidone, propranolol and gabapentin. The patient is right‐handed. During examination, we saw a bilateral position‐dependent and jerky action tremor, most severe when the arms were pronated (Video 1, part 1). There was dystonic posturing of the right hand, with hyperextension of the fingers, wrist flexion, ulnar deviation of the hand, as well as abnormal finger tapping. At rest, there was an intermittent tremor of the right index finger. A very mild, intermittent horizontal head tremor combined with subtle right‐sided rotation of the head was observed, suggestive of cervical dystonia. There was no bradykinesia, rigidity or ataxia. Polymyography showed tremulous activity with a frequency between 4.3 and 5.6 Hz (Fig. 1A). Brain magnetic resonance imaging (MRI) showed no lesions in the basal ganglia and cerebellum (Fig. 1B). Hence, she was diagnosed with idiopathic dystonic tremor syndrome.^1^ Six weeks later upon waking up in the morning, she noticed a minor weakness of the right leg, but also disappearance of tremor in the right arm. Computer tomography (CT) of the brain did not show any abnormalities in the acute setting, and she was started on antiplatelet therapy under suspicion of a stroke. MRI two weeks later showed a T2‐hyperintense lesion with diffusion restriction on diffusion‐weighted imaging, in the posterior limb of the internal capsule, suggestive of a stroke in the vascular territory of the anterior choroidal artery (Fig. 1B, C).

**Video 1:** In part 1 a bilateral position‐dependent and jerky action tremor is noted, most severe when the arms are pronated. There is dystonic posturing of the right hand. In part 2 (after stroke) the tremor on the right arm has disappeared. Hyperreflexia and a Babinski reflex, and subtle dysmetria during finger‐chase and heel‐to‐shin tests are noted on the right bodyside, indicating involvement of the pyramidal tracts. Dystonic posturing of the right arm is unchanged.

During a second visit to our outpatient clinic (Video 1, part 2) the tremor on the right arm had disappeared. Hyperreflexia and a Babinski reflex were noted on the right bodyside. A subtle dysmetria during finger‐chase and heel‐to‐shin tests was observed. Dystonic posturing at the right wrist remained unchanged, however dystonic posturing around the right thumb has improved. There was no more need for stereotactic surgery, the patient was able to perform all daily activities with her right hand to her satisfaction.

## Discussion

**Figure 2:**
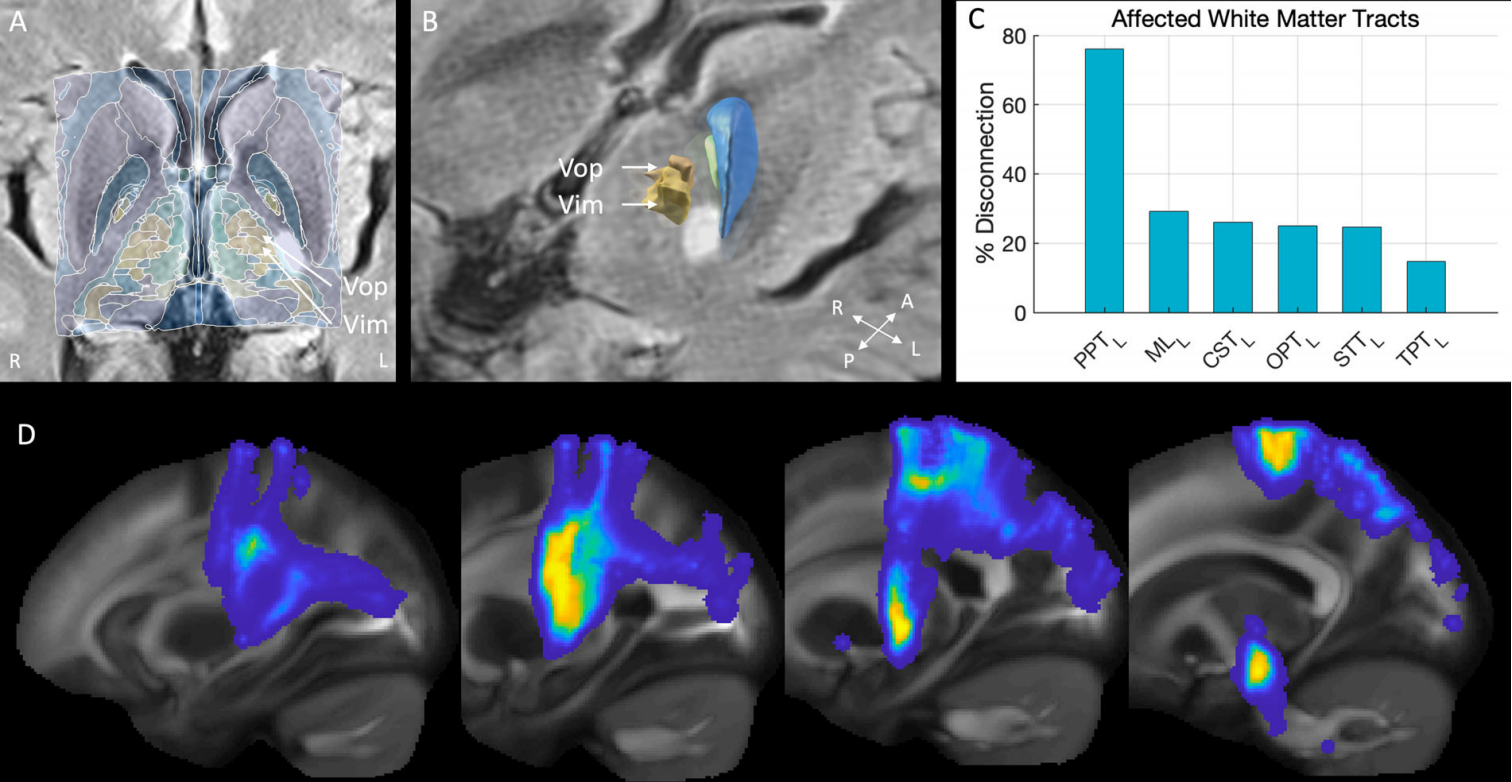
Atlas and tractography reconstructions of involved brain structures. (**A**) 2D axial reconstruction of the lesion in MNI space using the Lead‐DBS preprocessing pipeline and DISTAL subcortical atlas, showing proximity of the lesion to the Vim and VOp nucleus.^9^ Note that thalamo‐cortical radiations from these nuclei enter the posterior limb of the internal capsule in this region. Center of mass (MNI coordinates) of the lesion on DWI is x = −21.4, y = −16.5, z = 2.85 and on FLAIR x = −21.9, y = −17.1, z = 1.73. Lesion volume is 320 mm^3^ based on DWI and 374 mm^3^ based on FLAIR. (**B**) 3D view of the same reconstruction in MNI space showing the internal globus pallidus (green), external globus pallidus (blue), VOp and Vim nuclei (yellow). MRI images are displayed according to radiological convention (left = right). (**C**) A mask based on the lesion on DWI was used to assess white‐matter connectivity with the Lesion Quantification Toolbox.^10^ Percentage (%) disconnection refers to the amount of fiber bundles crossing the lesion. PPT_L_, parieto‐pontine tract, ML_L_, medial lemniscus, CST_L_, corticospinal tract, OPT_L_, occipito‐pontine tract, STT_L_, spinothalamic tract, TPT_L_, temporo‐pontine tract. (**D**) Coronal slices indicating involvement of mainly parieto‐pontine tracts using the same method as for Figure 2C. Please see the supplementary materials for further details on used methods and references. DWI and FLAIR‐based masks are available from the corresponding author, upon reasonable request.

This is the first report of disappearance of dystonic tremor after stroke. Using an atlas‐based reconstruction, we suggest that in this case a lesion in either the thalamocortical radiations exiting the thalamic ventralis intermediate (Vim) nucleus and ventral oralis posterior (VOp) nucleus, or the parieto‐pontine projections, might be responsible for attenuation of dystonic tremor (Fig. 2). This is in line with previous literature suggesting that any lesion within the cortico‐cerebello‐thalamo‐cortical circuit can disrupt tremor.^3^ Whether previously reported cases with essential tremor also had subtle signs of dystonia, and would at present be classified as dystonic tremor, is unknown, as for most of these cases video recordings are unavailable.

In 1953, Cooper already described the therapeutic effect of anterior choroidal artery ligation on Parkinsonian tremor.^4^ In a previous report by Dupuis and colleagues, a similar lesion as in our case is described in the posterior limb of the internal capsule in case 2, alleviating essential tremor, probably by involvement of thalamocortical projections.^5^ The Vim relays cerebellar input and the VOp relays pallidal input to the cortex. As can be seen in Figure 2A‐B, the lesion is in close proximity to both thalamic nuclei. A recent functional MRI study suggests that dystonic tremor involves both pallido‐thalamo‐cortical and cerebello‐thalamo‐cortical circuits.^6^ In contrast, in essential tremor abnormal activity in the cerebello‐thalamo‐cortical circuit, but not the basal ganglia, is observed.^7^ Furthermore, the optimal target for DBS in dystonic tremor appears to be at the border of the Vim, the conventional target for essential tremor, and the VOp.^8^ The lesion in our case might be ideally placed at the latero‐superior surface of the Vim and VOp nuclei, thereby interfering with abnormal activity in both circuits. An alternative explanation is that the lesion interrupted cortico‐pontine projections (Fig. 2C‐D), which relay cortical activity to the cerebellum via the pons. Interestingly, right‐sided dysmetria as observed in our case might also be explained by involvement of these projections, as this will also interfere with activity in the cortico‐cerebello‐thalamo‐cortical circuit.

In conclusion, we report disappearance of dystonic tremor after a stroke in the contralateral posterior limb of the internal capsule. We hypothesize involvement of the thalamocortical radiation or cortico‐pontine tracts. In either case, the common factor is disruption of the cortico‐cerebello‐thalamo‐cortical circuit, as has been described for other tremor disorders.

## Author Roles

(1) Research project: A. Conception, B. Organization, C. Execution; (2) Statistical Analysis: A. Design, B. Execution, C. Review and Critique; (3) Manuscript: A. Writing of the first draft, B. Review and Critique.

A.W.G.B.: 1A, B, C, 3A.

A.S.: 1C, 3B.

R.H.: 1A, 1B, 3B.

## Disclosures

**Ethical Compliance Statement:** The authors confirm that approval of an institutional review board was not required for this work. Informed consent from the patient is obtained. We confirm that we have read the Journal's position on issues involved in ethical publication and affirm that this work is consistent with those guidelines.

**Funding Sources and Conflicts of Interest:** The authors declare that there are no funding sources or conflicts of interest to report.

**Financial Disclosures for the Previous 12 Months:** Rick Helmich was funded by the Netherlands Organization for Scientific Research (VIDI grant, #09150172010044), the Michael J. Fox Foundation (MJFF‐021001 and #15581), and an EU‐JPND grant (#10510062110006). Arthur Buijink was funded by travel stipends from the Dr. Jan Meerwaldt Stichting and the Remmert Adriaan Laan fonds.

## Supporting information


**Supplementary material**: Supplementary methods regarding atlas and tractography reconstructions.
