# Supplementary material for: Dystonic Tremor Disappearance after Internal Capsule Stroke
Source: Mov Disord Clin Pract. 2023 May 31;10(8):1203–6. doi: 10.1002/mdc3.13790 (PMC10450227; doi:10.1002/mdc3.13790)
Supplement: Supplementary file 1 — Supplementary material: Supplementary methods regarding atlas and tractography reconstructions. [file MDC3-10-1203-s001.docx]

**Supplementary material**

Reconstructions of the lesion in MNI space were made using the Lead-DBS preprocessing pipeline (version 2.6, Horn et al. 2019). DICOM images were converted to the NIfTI file format and linearly co-registered to the T1 sequence using SPM12 (Friston 2011; <http://www.fil.ion.ucl.ac.uk/spm/software/>). Scans were spatially normalized into MNI_ICBM_2009b_NLIN_ASYM space (Fonov et al. 2011) using the SyN registration approach as implemented in Advanced Normalization Tools (Avants et al. 2008; http://stnava.github.io/ANTs/). Nonlinear deformation into template space was achieved in five stages: After two linear (rigid followed by affine) steps. A nonlinear (whole brain) SyN-registration stage was followed by two nonlinear SyN-registrations that consecutively focused on the area of interest as defined by subcortical masks (Schoenecker 2008). The DISTAL Atlas was used for 2D and 3D visualization (Ewert et al. 2017).

Subsequently a mask based on the lesion on DWI was used to assess white-matter connectivity with the Lesion Quantification Toolbox (Griffis et al. 2021). Tractography data is derived from 70 tracts in the HCP-842 tractography atlas (Yeh et al. 2018). Percentage (%) disconnection refers to the amount of fiber bundles crossing the lesion.

Supplementary references

Avants, B. B., Epstein, C. L., Grossman, M., & Gee, J. C. (2008). Symmetric diffeomorphic image registration with cross-correlation: evaluating automated labeling of elderly and neurodegenerative brain. Medical Image Analysis, 12(1), 26-41. DOI: 10.1016/j.media.2007.06.004

Ewert, S., Plettig, P., Li, N., Chakravarty, M. M., Collins, D. L., Herrington, T. M., et al. (2017). Toward defining deep brain stimulation targets in MNI space: A subcortical atlas based on multimodal MRI, histology and structural connectivity. NeuroImage;170:271-282. DOI: 10.1016/j.neuroimage.2017.05.015.

Fonov VS, Evans AC, Botteron K, Almli CR, McKinstry RC, Collins DL and Brain Development Cooperative Group (2011). Unbiased average age-appropriate atlases for pediatric studies, NeuroImage, Volume 54, Issue 1, DOI: 10.1016/j.neuroimage.2010.07.033

Friston, K. J., Ashburner, J. T., Kiebel, S. J., Nichols, T. E., & Penny, W. D. (2011). Statistical Parametric Mapping: The Analysis of Functional Brain Images. Academic Press.

Griffis JC, Metcalf N v., Corbetta M, Shulman GL. Lesion Quantification Toolkit: A MATLAB software tool for estimating grey matter damage and white matter disconnections in patients with focal brain lesions. Neuroimage Clin.; 2021;30.

Horn, A., Li, N., Dembek, T. A., Kappel, A., Boulay, C., Ewert, S., Tietze, A., Husch, A., Perera, T., Neumann, W.-J., Reisert, M., Si, H., Oostenveld, R., Rorden, C., Yeh, F.-C., Fang, Q., Herrington, T. M., Vorwerk, J., & Kühn, A. A. (2019). Lead-DBS v2: Towards a comprehensive pipeline for deep brain stimulation imaging. NeuroImage, 184, 293–316. DOI: 1016/j.neuroimage.2018.08.068

Schoenecker, T., Kupsch, A., Kuehn, A. A., Schneider, G.-H., & Hoffmann, K. T. (2009). Automated Optimization of Subcortical Cerebral MR Imaging-Atlas Coregistration for Improved Postoperative Electrode Localization in Deep Brain Stimulation. AJNR Am J Neuroradiol, 30(10), 1914-1921. DOI: 10.3174/ajnr.A1741.

Yeh FC, Panesar S, Fernandes D, Meola A, Yoshino M, Fernandez-Miranda JC, et al. Population-averaged atlas of the macroscale human structural connectome and its network topology. Neuroimage.; 2018;178:57–68.
